# Herpes Simplex Virus Type 1 Neuronal Infection Triggers the Disassembly of Key Structural Components of Dendritic Spines

**DOI:** 10.3389/fncel.2021.580717

**Published:** 2021-02-23

**Authors:** Francisca Acuña-Hinrichsen, Adriana Covarrubias-Pinto, Yuta Ishizuka, María Francisca Stolzenbach, Carolina Martin, Paula Salazar, Maite A. Castro, Clive R. Bramham, Carola Otth

**Affiliations:** ^1^Institute of Clinical Microbiology, Faculty of Medicine, Universidad Austral de Chile, Valdivia, Chile; ^2^Center for Interdisciplinary Studies on the Nervous System (CISNe), Universidad Austral de Chile, Valdivia, Chile; ^3^Post-graduate Program, Science Faculty, Universidad Austral de Chile, Valdivia, Chile; ^4^Institute of Biochemistry II, Goethe University School of Medicine, Frankfurt am Main, Germany; ^5^Department of Biomedicine, University of Bergen, Bergen, Norway; ^6^School of Medical Technology, Austral University of Chile, Puerto Montt, Chile; ^7^Institute of Biochemistry and Microbiology, Faculty of Science, Universidad Austral de Chile, Valdivia, Chile; ^8^Janelia Research Campus, HHMI, VA, United States

**Keywords:** Herpes Simplex Virus Type 1 (HSV-1), neurodegeneration, neurotropic virus, Arc protein, memory consolidation, dendritic spines

## Abstract

Herpes simplex virus type 1 (HSV-1) is a widespread neurotropic virus. Primary infection of HSV-1 in facial epithelium leads to retrograde axonal transport to the central nervous system (CNS) where it establishes latency. Under stressful conditions, the virus reactivates, and new progeny are transported anterogradely to the primary site of infection. During the late stages of neuronal infection, axonal damage can occur, however, the impact of HSV-1 infection on the morphology and functional integrity of neuronal dendrites during the early stages of infection is unknown. We previously demonstrated that acute HSV-1 infection in neuronal cell lines selectively enhances Arc protein expression - a major regulator of long-term synaptic plasticity and memory consolidation, known for being a protein-interaction hub in the postsynaptic dendritic compartment. Thus, HSV-1 induced Arc expression may alter the functionality of infected neurons and negatively impact dendritic spine dynamics. In this study we demonstrated that HSV-1 infection induces structural disassembly and functional deregulation in cultured cortical neurons, an altered glutamate response, Arc accumulation within the somata, and decreased expression of spine scaffolding-like proteins such as PSD-95, Drebrin and CaMKIIβ. However, whether these alterations are specific to the HSV-1 infection mechanism or reflect a secondary neurodegenerative process remains to be determined.

## Introduction

Herpes simplex virus type 1(HSV-1) is a neurotropic double-stranded DNA virus with a high worldwide prevalence. After primary infection in buccal mucosa epithelium, viral progeny can be transported retrogradely through the axons of sensory neurons to the central nervous system (CNS) (Otth et al., 2016). These virions can establish a persistent latent infection in the brain of infected individuals, repressing gene expression to a latency-associated microRNA called LAT. Viral reactivation can occur under stressful conditions, and the production of viral progeny alters protein synthesis and cellular homeostasis (Cliffe and Wilson, 2017). HSV-1-infection is associated with neurodegenerative processes (Eimer et al., 2018; Readhead et al., 2018). Although damage to the axonal cytoskeleton has been reported (Zambrano et al., 2008), no studies have evaluated the impact of HSV-1 infection on the morphology or function of the postsynaptic compartment and dendritic spines of excitatory synapses.

Synapses are the basic unit of neuronal communication and their disruption is associated with many neurodegenerative diseases (Lei et al., 2016). Excitatory synapses use glutamate as the main neurotransmitter in the brain where the majority of the synaptic connections between the glutamatergic neurons are made on dendritic spines (Stefen et al., 2016). These structures are composed of actin cytoskeleton, receptors, and scaffolding proteins that maintain the shape and function of these protrusions. Activity-dependent changes require *de novo* protein synthesis, where one of the most important immediate early genes (IEGs) that is rapidly up-regulated after synaptic activity is Activity-Regulated Cytoskeleton-Associated Protein: Arc. Arc is an essential element for multiple forms of protein synthesis-dependent plasticity, including long-term potentiation (LTP), long-term depression (LTD) and related homeostatic synaptic scaling (Guzowski et al., 2000; Plath et al., 2006; Shepherd et al., 2006; Messaoudi et al., 2007; Bramham et al., 2010; Korb and Finkbeiner, 2011).

Arc interacts with NMDARs (N-methyl-D-aspartic acid receptors) and PSD-95 at the postsynaptic density, forming receptor adhesion signaling complexes in lipid rafts (Delint-Ramirez et al., 2010; Collins et al., 2017). In LTP consolidation, SUMOylated Arc forms a complex with Drebrin, a major regulator of cytoskeletal dynamics in dendritic spines (Nair et al., 2017). Even though Arc expression is induced by synaptic activity, the protein is targeted to inactive spines by association with CaMKIIβ (Okuno et al., 2012; El-Boustani et al., 2018). CaMKIIβ binds Arc more tightly in the absence of Ca^2+^/Calmodulin (CaM), and therefore, in the absence of T287 autophosphorylation (Okuno et al., 2012). Thus, Arc protein interacts with distinct protein partners to participate in the regulation of multiple forms of synaptic plasticity (Nikolaienko et al., 2018). Interestingly, we previously demonstrated that HSV-1 acute infection increases Arc mRNA and protein expression in different neuronal cell lines, and the up-regulation is dependent on an active viral replicative cycle (Acuña-Hinrichsen et al., 2019). Nevertheless, the impact of HSV-1 infection on dendritic spines, dendritic arborization and signaling is unknown.

Here we show that HSV-1 results in Arc upregulation combined with downregulation in the expression of structural proteins (CaMKIIβ, Drebrin and PSD-95) in the post-synaptic compartment. This downregulation is associated with neurite retraction and reduced dendritic arborization, and loss of glutamate induced calcium transients, in infected neurons. We also found that extracellular signal-regulated kinase (Erk) activation is not involved in HSV-1-induced phenotypes. Altogether, our results demonstrate progressive changes in synaptic-dendritic structure and function in HSV-1 infected neurons. This mechanism of postsynaptic disassembly may contribute to the development of focal neuronal damage resulting from successive reactivation episodes of infected individuals.

## Results

### HSV-1 Induces Morphological Changes in Cortical Neurons

HSV can trigger or block apoptosis in a cell-dependent manner (Galvan and Roizman, 1998; Perkins et al., 2003; Esaki et al., 2010). The infection and apoptosis rate in primary cultures infected with HSV-1 has a typical cytopathic effect during the late stages of infection (18-24 h post-infection hpi) (Leyton et al., 2015; Martin et al., 2017); however, the apoptosis rates during the early infection stage are unknown. Therefore, we focused on the early stages of HSV-1 infection (up to 8 h). For this, primary cortical neurons from 14 to 21 DIV (days *in vitro*) were infected at an infection multiplicity of 5 (MOI = 5).

**FIGURE 1:**
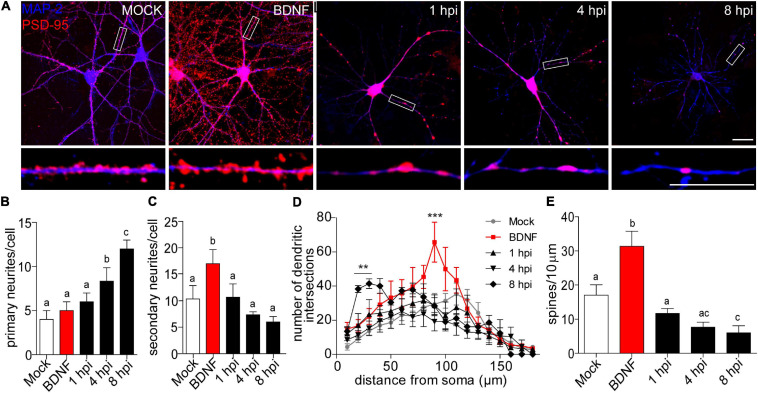
Morphological analyses of dendritic complexity in HSV-1 infected neurons. **(A)** Representative images of cortical neurons infected with HSV-1 MOI 10, BDNF was used as a positive control. Antibodies against PSD-95 and MAP-2 are shown in blue and red, respectively. Scale bar in = 20 μm. **(B)** Average number of primary neurites **(C)** average number of secondary neurites. **(D)** The dendritic arborization was calculated using Sholl analysis and **(E)** the quantification of spines was represented as the number of spines/10 μm. The quantifications are representative of three independent experiments. Statistical significance was determined by One-Way ANOVA, followed by Tukey’s test. Bars represent the mean ± SD of biological replicates (10 neurons per treatment). Different letters above the mean bars apply to significant differences between groups *p* < 0.05. Sholl analysis statistical significance was determined by Two-Way ANOVA followed by Bonferroni’s test. ***p < 0.001; **p < 0.01.

We performed classic morphological analyses during infection kinetics of 1, 4, 8 hpi to quantify dendritic spine remodeling. We used MAP-2 immunostaining to label dendritic arbors combined with PSD-95 staining to quantify dendritic spine density. Two hours of BDNF treatment was used as a positive control for synaptic protein expression and arborization (Wibrand et al., 2012; Leal et al., 2014; Figures 1A–E). We observed a decrease in neuritic immunoreaction of these markers starting at 4 hpi compared to mock-infected and BDNF-treated neurons. This phenotype became clearer at 8 hpi where neurons had thin neuritic processes and shrunken phenotypes (Figure 1A). The prevalent reduction of microtubular structures detected by MAP-2 staining was previously reported as one of the effects of HSV-1 in neuronal microtubule dynamics (Zambrano et al., 2008).

A morphologically functional neuron is defined by the length and number of branches in its neuritic processes. In neurodegenerative states, these processes are interrupted or shortened (Chevalier-Larsen and Holzbaur, 2006; Dugger and Dickson, 2017). Here we provide evidence that HSV-1 induces a specific neuronal phenotype –a shrunken dendritic arbor. Processes extending from the cell body are defined as primary neurites, while those that start from primary neurites are secondary neurites (Langhammer et al., 2010). We quantified dendritic arborization in terms of the average number of primary and secondary neurites (Figures 1B,C). There was no difference in the average number of primary dendrites, when comparing the mock control, BDNF treatment and the 1 hpi group (average of 4.5 primary neurites per neuron). Interestingly, at 4 and 8 hpi the average number of primary neurites was significantly higher (average of ∼8 and 12, respectively Figure 1B). As expected, the BDNF control was significantly increased ∼2-fold in secondary neurites compared to the mock control. However, there were no differences between the average number of secondary neurites of the mock control and the infected neurons at 1, 4, and 8 hpi (Figure 1C). The immunocytochemistry shown in Figure 1A together with the observation of the increased average number of primary neurites in infected neurons reflects the remodeling of the cytoarchitecture of the infected neurons in a pathological way. Neurons with shrunken arbors are less likely to establish connections within their network (Kerrigan and Randall, 2013).

Sholl analysis confirmed the retraction phenomena, where the number of dendritic intersections was higher closer to the somata rather than in distal neurites, with a mean of 41.4 intersections at 30 μm from the somata center in 8 hpi neurons (Figure 1D). The dendritic spine density was quantified by immunoblot analyses of PSD-95 expression. Spine density was significantly reduced by 65% at 8 hpi compared to mock-infected neurons, suggesting a structural defect at a very specific level of neuronal complexity.

### HSV-1 Affects the Expression and Distribution of Dendritic Spine Proteins in Cortical Neurons

Dendritic spines are the small, protruding, subcellular compartments found on the dendritic processes of neurons where the majority of excitatory synaptic signaling occurs in the brain (McGuier et al., 2019). Spines contain a postsynaptic density (PSD), the electron-dense thickening on the spine head that consists of receptors, ion channels, and signaling systems involved in synaptic transmission. Underneath the receptors, several scaffolding proteins are involved in the maintenance and shape of these protrusions (Koganezawa et al., 2017). Due to the evident morphological alteration in the infected neurons during the early stages of infection, we evaluated the expression and subcellular distribution of Arc, CaMKIIβ, Drebrin, and PSD-95; known to be resident proteins of the dendritic spines showing higher expression levels during synaptic activity in healthy neurons.

**FIGURE 2:**
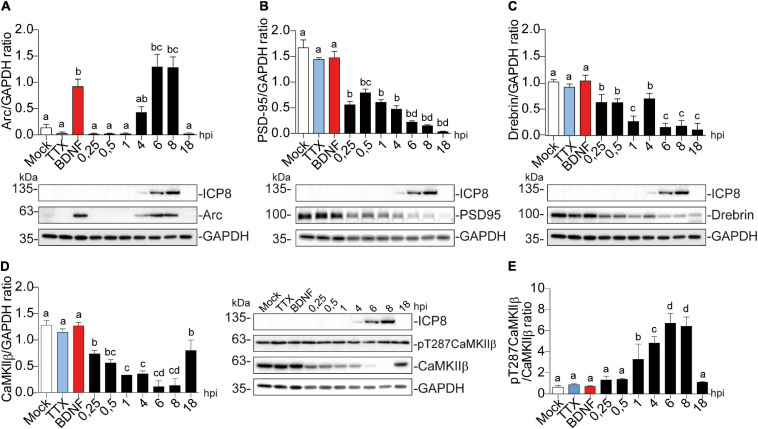
Arc protein expression is increased in cortical neurons infected with HSV-1, while CaMKIIb, Drebrin, and PSD-95 expression are decreased. Quantification of immunoblot analyses showing **(A)** Arc and its dendritic binding partners: **(B)** PSD-95, **(C)** Drebrin, **(D)** CaMKIIβ (left panel) and pT287 CaMKIIβ (right panel) during infection kinetics with HSV-1 (Mock, 0.25; 0.5; 1, 4, 6, 8, and 18 hpi). BDNF and TTX are used as positive and negative controls of Arc induction, respectively. After gel scanning and densitometry quantitation, the results were expressed as the ratio of protein levels normalized to total GAPDH. Statistical significance was determined by One-Way ANOVA, followed by Tukey’s test. Bars represent the mean ± SD of biological replicates. Different letters above the mean bars apply to significant differences between groups *p* < 0.05. The blots are representative of three independent experiments.

All HSV-1 infection effects were compared to mock-infected cells as a negative control (Figures 2A–D). 2 h of TTX treatment was used as a negative control treatment since it inhibits basal neuronal activity (Okuno et al., 2012). BDNF was used a positive control for the proteins studied (El-Sayed et al., 2011; D. Y. Chen et al., 2012; Huang et al., 2013; Nair et al., 2017). HSV-1-infected neurons significantly increased Arc immunodetection, present at 6 and 8 hpi and lost by 18 hpi (Figure 2A).

A similar temporal expression pattern was observed for ICP8 (single-strand DNA [ssDNA]-binding protein), an essential HSV-1 replication protein (Weller et al., 2014) that is used as an infection marker (Figures 2A–D). Contrary to Arc, the expression of PSD-95, Drebrin and CaMKIIβ progressively decreased; starting from the first time point (15 min after infection = 0.25 hpi) (Figures 2B–D).

**FIGURE 3:**
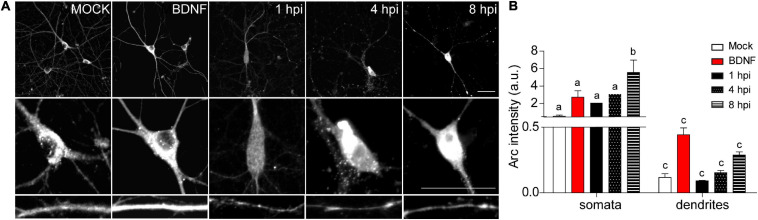
HSV-1-induced Arc is enriched in neuronal somata rather than dendrites of infected neurons. **(A)** Immunocytochemical analyses of Arc protein subcellular distribution during infection kinetics. Middle and low panels show magnifications of somata and dendrites. Scale bars: 20 μm **(B)** Quantification of Arc fluorescence intensity in HSV-1 infected neurons versus positive (BDNF) and negative (Mock) controls. Statistical significance was determined by One-Way ANOVA, followed by Tukey’s test. Bars represent the mean ± SD of biological replicates (10 neurons per treatment). Different letters above the mean bars apply to significant differences between groups *p* < 0.05. The images and quantifications are representative of three independent experiments.

Auto-phosphorylated CaMKIIβ levels increased significantly from 1 to 8 hpi compared to mock-infected neurons (Figure 2D). This process happens in the presence of Ca^2+^ and Calmodulin (CaM) (Fink and Meyer, 2002), suggesting that elevated intracellular Ca^2+^ levels could be responsible for CaMKIIβ autophosphorylation during HSV-1 infection. Consistently, previous work showed high intracellular Ca^2+^ concentrations in cortical neurons upon 18-24 hours of HSV-1 infection (Cheshenko et al., 2003, 2013). Arc is not detected in presynaptic terminals or axons after an increase in synaptic activity, but is highly expressed in dendrites (Fujimoto et al., 2004; Rodriguez et al., 2005), the postsynaptic density (Husi et al., 2000; Moga et al., 2004; Rodriguez et al., 2005) and the nucleus (Irie et al., 2000; Bloomer et al., 2007). We have already demonstrated that Arc protein expression is increased during HSV-1 infection in several neuronal cell lines. Thus, we also investigated Arc subcellular distribution in somata versus dendrites of infected cortical neurons (Figures 3A,B). We selected 10 different regions of interest (ROIs) in both somata and dendrites of mock-infected, BDNF-treated and 1, 4, and 8 hpi of HSV-1 (20 neurons/3 repetitions), normalized by area. Arc is significantly increased at 8 hpi in the somata of the infected neurons compared to the mock control (Figure 3B). Thus, HSV-1 infection of cortical neurons results in a massive acute (8 hours) increase in Arc expression which is 4 fold higher in somata relative to dendrites.

**FIGURE 4:**
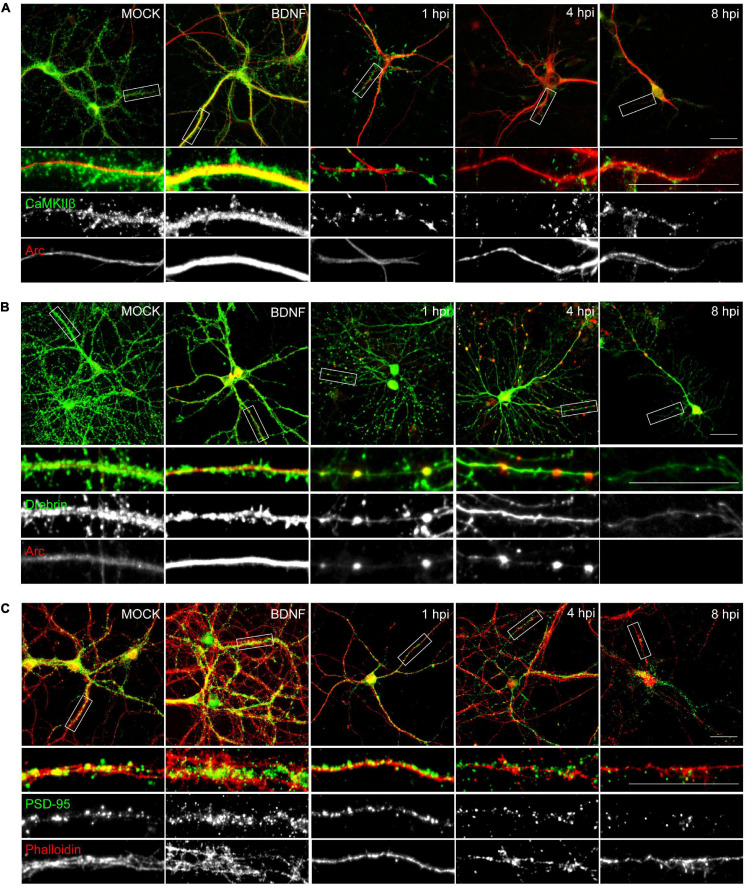
HSV-1 neuronal infection alters the normal distribution of Arc, CaMKIIβ, Drebrin and PSD-95. Immunocytochemistry analyses of infection kinetics. BDNF was used as a positive control for synaptic protein expression. Double staining against **(A)** Arc and CaMKIIβ; **(B)** Arc and Drebrin; **(C)** PSD-95 and cytoskeleton probe: Phalloidin (red). Secondary antibodies coupled to Alexa Fluor 488 and 568. Bars: 20 mm. The results shown are representative of three independent experiments.

The morphological alterations (Figure 1) together with the decrease in synaptic protein levels of the infected neurons (Figures 2, 3), was supported by immunocytochemical methods (Figure 4). Actin cytoskeletal structure was detected by a fluorescent phalloidin probe which binds filamentous actin. All the proteins analyzed, except for Arc, exhibited a decrease in relative fluorescence intensity from 1 hpi (Figures 4A–C), in contrast to the mock-infected and BDNF-treated neurons, which showed normal protein dendritic localization and filamentous actin labeling.

### Synaptoneurosome Content of HSV-1 Infected Neurons

**FIGURE 5:**
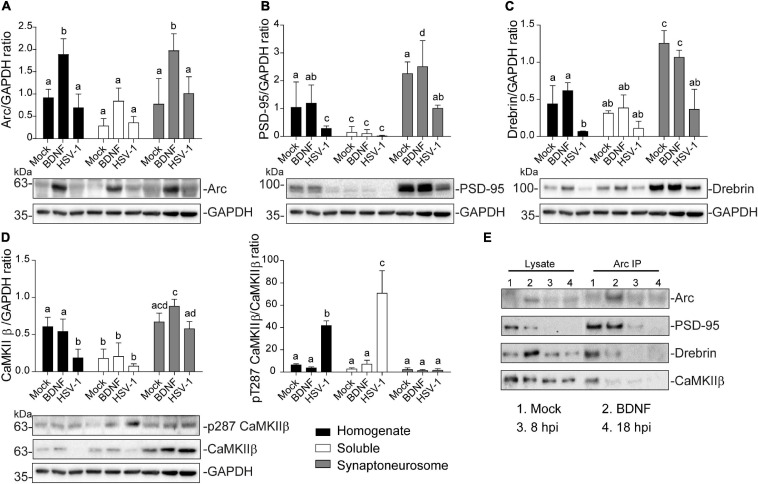
Altered protein content in synaptoneurosomes of HSV-1 infected cortical neurons. Quantification of immunoblot analyses of synaptoneurosomal isolation HSV1-infected and BDNF-stimulated cortical neurons, showing **(A)** Arc, **(B)** PSD-95, **(C)** Drebrin **(D)** CaMKIIβ and its activation through T287 phosphorylation. GAPDH was used to normalize each blot. **(E)** Immunoprecipitation of Arc from the synaptoneurosomal fraction from Mock, BDNF-stimulated and HSV-1 -infected neurons at 8 and 18 hpi. Immunoblot against dendritic binding partners: PSD-95, Drebrin CaMKIIβ, and β-actin. After gel scanning and densitometry quantitation, the results were expressed as the ratio of protein levels normalized to total GAPDH. Statistical significance was determined by One-Way ANOVA, followed by Tukey’s test. Bars represent the mean ± SD of biological replicates. Different letters above the mean bars apply to significant differences between groups *p* < 0.05. The blots are representative of three independent experiments.

To further assess and quantify changes in the protein content in dendritic spines we used fractionated synaptoneurosomes. The term synaptoneurosome refers to entities in which a presynaptic bouton (synaptosome) is attached to a resealed postsynaptic spine (neurosome) (Villasana et al., 2006). This means that the isolated synaptoneurosomes should have membrane and cytosolic proteins of the dendritic spines. In this way, we isolated synaptoneurosomes from mock-infected neurons, BDNF-treated, and infected with HSV-1 at 8 hpi (critical time point of the infected neuron phenotype). By using a low osmolarity buffer we effectively obtained 3 fractions after serial filtration and centrifugation steps (Villasana et al., 2006). Figures 5A–D shows immunoblot analyses of each obtained fraction. Total homogenate (obtained with low osmolarity buffer), the soluble fraction (supernatant after filtration and centrifugation steps), and the synaptoneurosomal fraction (pellet resuspended in RIPA buffer) were obtained for each treatment (Ishizuka and Bramham, 2020)

Arc protein expression was significantly enriched in the synaptoneurosomal fraction of BDNF-treated neurons compared to the total homogenate and soluble fractions (Figure 5A). However, there were no significant differences between the HSV-1-infected neurons in any of the obtained fractions, compared to the mock control (Figure 5A). The fact that we did not observe highly concentrated Arc in any of the fractions obtained from HSV-1-infected neurons, might be due to Arc oligomerization properties. Arc is capable of self-oligomerization forming a stable complex (Myrum et al., 2015; Eriksen et al., 2020) which cannot be dissolved by a low osmolarity buffer such as the one used in this synaptoneurosomal purification. Many of the stable protein complexes, like those found in synaptic machinery (Chen et al., 1999) can be SDS-resistant. Interestingly, we also found a high molecular weight complex in an SDS gel following infection, suggesting that Arc oligomers might form an SDS-resistant complex (Supplementary Figure 1).

As expected in non-infected neurons PSD-95, Drebrin, and CaMKIIβ were enriched in the synaptoneurosomal fraction compared to the soluble fraction and the total homogenate (Figures 5B–D). In HSV-1-infected neurons, PSD-95 and Drebrin expression were significantly decreased in both synaptoneurosomes and the total homogenate (Figures 5B,C). CaMKIIβ expression levels were also decreased in HSV-1-infected neurons in the total homogenate fraction, but not in the synaptoneurosomal fraction, relative to the mock control (Figure 5D left panel). Autophosphorylation of CaMKIIβ was highly increased in the total homogenate and soluble fractions, compared to the synaptoneurosomal fraction (Figure 5D right panel).

Since Arc is known to act as a hub protein, we proposed that HSV-1 infection could result in altered Arc protein-protein interactions in dendritic spines. To address this hypothesis, we performed Arc immunoprecipitation in the synaptoneurosome samples and immunoblot for PSD-95, Drebrin and CaMKIIβ along with Arc itself (Figure 5E). Co-immunoprecipitation of Arc binding partners was detected in mock-infected and BDNF-treated neurons. At 8 hpi, interaction with Drebrin and CaMKIIβ was reduced compared to the mock control, and at 18 hpi, no interaction with any of the studied proteins was found. These results complement the observation of morphological disruption in infected cortical neurons, which is accompanied by an acute and progressive decline of Arc protein-protein interactions in spines.

### Infected Neurons Exhibit an Altered Response to Glutamate Stimulation

Glutamate mediates excitatory neurotransmission and is the major neurotransmitter in the central nervous system (Marx et al., 2015). Glutamate acts through two receptor families: metabotropic and ionotropic receptors (Simeone et al., 2004). Ionotropic glutamate receptors are ligand-gated ion channels, which are classified into three subtypes according to their most selective agonist: N-methyl-D-aspartic acid (NMDA), α-amino-5-hydroxy-3-methyl-4-isoxazole propionic acid (AMPA), and kainate. The metabotropic receptors are G protein (guanine nucleotide-binding protein)-coupled receptors linked to second-messenger systems. Some are linked to phospholipase C, resulting in the release of intracellular calcium, whereas others are negatively linked to adenylate cyclase (Marx et al., 2015). Exposure of neurons to glutamate increases the concentration of free intracellular Ca^2+^ (MacDermott et al., 1986; Schmolesky et al., 2002).

**FIGURE 6:**
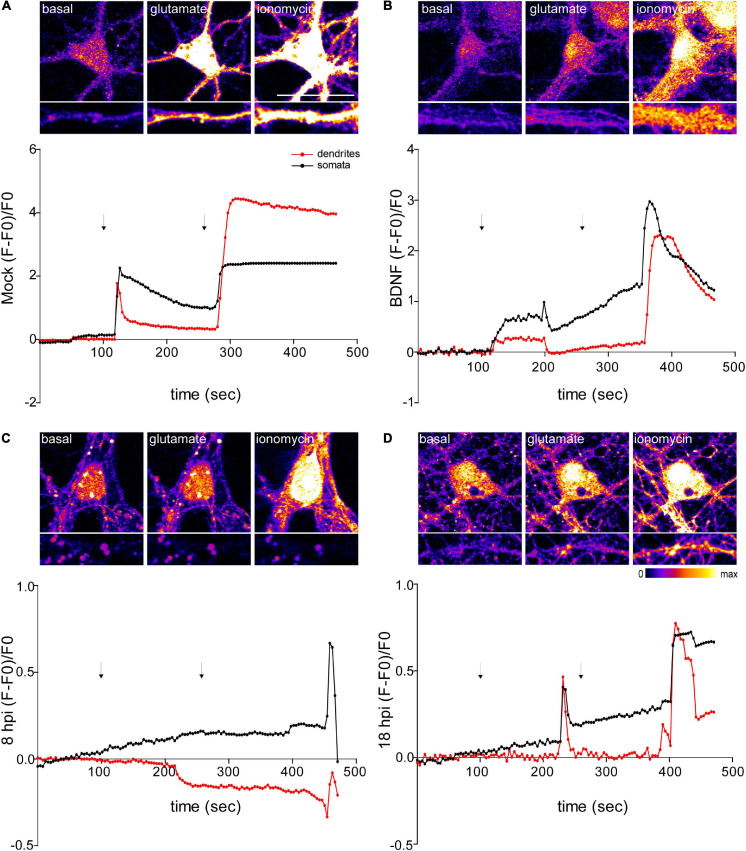
Infected neurons show an impaired response to glutamate stimulation. Top panels show representative microscopy images of somata and dendrites. **(A)** Mock-infected, **(B)** BDNF-treated and HSV-1-infected neurons at 8 and 18 hpi, respectively (**C** and **D**). Variations of relative cytosolic Ca^2+^ concentrations were monitored by fluorescence in Fluo4-loaded cortical neurons. The first arrow indicates the addition of glycine plus glutamate. The second arrow indicates the addition of the positive control ionomycin. The data is representative of traces of three independent experiments.

In neurons, intracellular calcium plays a critical role in the induction of synaptic activity and activation of signaling pathways. Several reports have shown that increased neuronal excitability and synaptic activity that lead to dysregulation of intracellular Ca^2+^ signaling and homeostasis are strongly related to neurodegenerative cellular mechanisms such as those observed in Alzheimer‘s disease (AD) (Paula-Lima et al., 2013; Piacentini et al., 2015a; Harris and Harris, 2018). To evaluate the response of the infected neurons to glutamate, we used a calcium-sensitive probe Fluo-4-AM as an indicator of calcium changes. We measured intracellular Ca^2+^ changes in live HSV-1 infected cortical neurons, and fluorescence intensity traces were generated for the ROIs selected in somata and dendrites separately (5 neurons per treatment/3 repetitions) (Figure 6).

NMDA receptors require the binding of glycine and glutamate, in combination with the release of a voltage-dependent magnesium block to open an ion conductive pore across the membrane bilayer (Lee et al., 2014). Thus, we recorded fluorescence intensity levels before and after application of glycine + glutamate to live neurons. As a positive control for Ca^2+^ entry we used ionomycin, an ionophore that raises intracellular Ca^2+^ levels. As expected, mock control and BDNF-treated neurons elicited a rise in fluorescence intensity after the addition of glycine + glutamate (∼20 s after the addition) (Figures 6A,B). However, the fluorescence intensity of infected neurons did not increase at 8 or 18 hpi after glutamate stimulation (Figures 6C,D). Instead, 18 hpi neurons showed a delayed peak in fluorescence intensity. It has been previously demonstrated that HSV-1 infection increases intracellular Ca^2+^ (Piacentini et al., 2011,2015). Sustained increases of nuclear Ca^2+^ are related with IEG expression, but could also reflect a pre-apoptotic state (Bading, 2013). The structural collapse of infected neurons at dendritic level indicates that HSV-1 is not only causing structural dendritic damage, but also altering physiological response to glutamatergic neurotransmission of neurons, which is critical to their biological function. Overall, all the alterations caused by HSV-1 acute infection in neuronal cells previously shown by us and others (Martin et al., 2017; Duarte et al., 2019), support the role of HSV-1 as a neurodegeneration risk factor.

### HSV-1 Activates CREB in the Early Stages of Infection

Immediate early gene transcription can be induced by the activation of various extracellular receptors and intracellular pathways and many of them converge in the activation of extracellular signal-regulated kinase (Erk). Within the Arc gene, there are regulatory elements that interact with transcription factors downstream of Erk signaling. The Arc gene has a synaptic activity-responsive element (SARE) which contains binding sites for CREB (cAMP response element-binding protein) and other transcriptional regulators, including SRF (serum response factor) and MEF2 (myocyte enhancer factor-2) (Kawashima et al., 2009). To explore the signaling pathway involved in increased Arc expression in HSV-1 infected neurons, we measured protein phosphorylation levels of Erk and the downstream target CREB across the time course of infection as shown in Figure 2.

**FIGURE 7:**
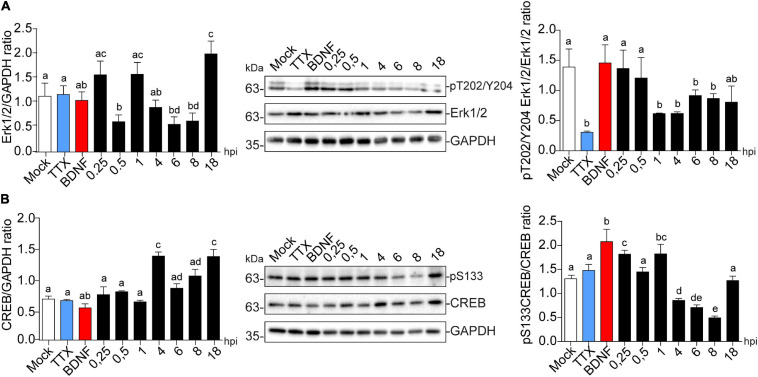
HSV-1 induces CREB phosphorylation in the early stages of neuronal infection. Immunoblot analyses showing **(A)** Erk1/2 total protein expression (left panel) and phosphorylated levels in T202/Y204 (right panel) and **(B)** CREB total protein expression (left panel) and phosphorylated levels in S133 during HSV-1 infection kinetics (Mock, 0.25; 0.5; 1, 4, 6, 8, and 18 hpi), BDNF and TTX were used as positive and negative controls for signaling pathway activation and inhibition, respectively. Statistical significance was determined by One-Way ANOVA, followed by Tukey’s test. Bars represent the mean ± SD of biological replicates. Different letters above the mean bars apply to significant differences between groups *p* < 0.05. The blots are representative of three independent experiments.

Immunoblot detection showed that Erk1/2 expression levels were reduced at 0.5, 6, and 8 hpi, to then recover and significantly increase compared to the mock control (Figure 7A left panel). Erk1/2 phosphorylation at T202/Y204 was significantly decreased from 1 to 8 hpi, which may reflect deactivation of the pathway at this precise time (Figure 7A right panel). CREB levels were also significantly increased at 4 and 18 hpi (Figure 7B left panel), and its phosphorylation levels at S133 were up-regulated after only 15 min post-infection compared to the mock control followed by a decrease at 8 hpi (Figure 7B right panel). These results are complemented by the observed increase in phosphorylation levels of CREB starting at 1 hpi, which is maintained to 4 hpi in the HT22 cell line (Supplementary Figure 2A), where phospo-S133 CREB is translocated to the nucleus in infected cells (Supplementary Figure 2B). Altogether, these data suggest that increased Arc expression during viral infection could be associated with CREB activation.

### Dendritic Protein Levels During HSV-1 Neuronal Infection Are Regulated Through Erk-Independent Signaling

To find a critical point for dendritic protein alteration in HSV-1-infected neurons, we used the MEK (Erk kinase) inhibitor U0126 for 1 h at different time points along 8 h of infection. We treated neurons with U0126 for 1 h; before infection (1 hbi), simultaneously with infection (0126 + HSV-1), and during infection at 1, 3, 5, and 7 hpi. Extracts from BDNF-treated neurons were used as a positive control for Arc expression and extracts from 8 hpi neurons were used as a positive control for HSV-1-induced Arc expression and PSD-95, Drebrin, and CaMKIIβ decrease. We also incubated BDNF in presence of U0126 as an internal control.

**FIGURE 8:**
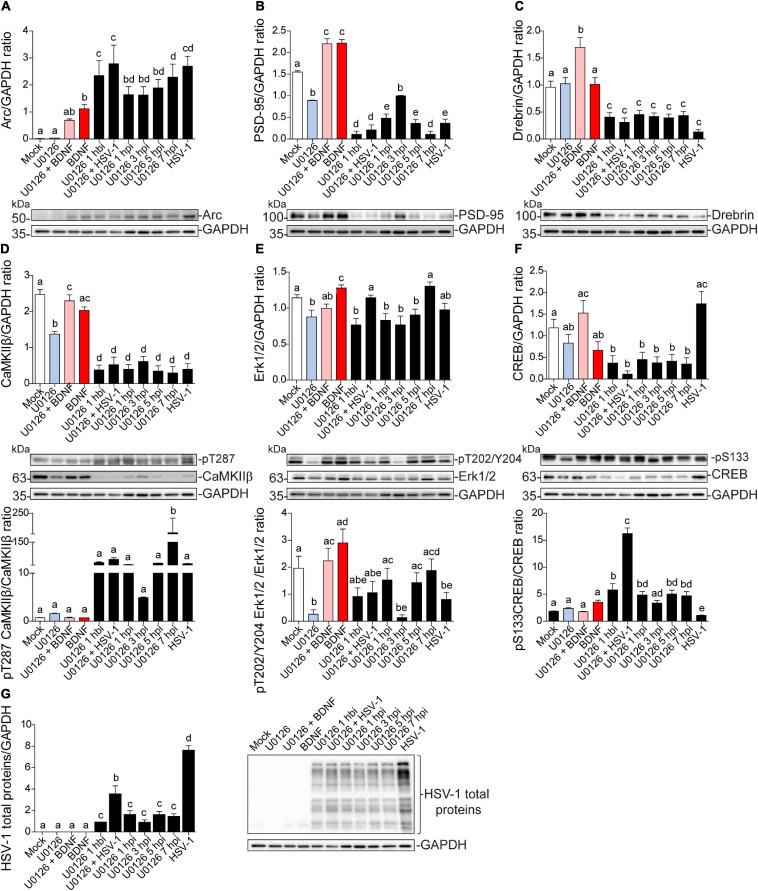
Dendritic protein levels during HSV-1 neuronal infection is controlled by Erk-independent signaling. Immunoblot analyses showing protein total levels at 8 hpi. **(A)** Arc, **(B)** PSD-95, **(C)** Drebrin, **(D)** CaMKIIβ, **(E)** Erk1/2, **(F)** CREB and, **(G)** HSV-1 total proteins. BDNF was used as a positive control of Arc expression and the U0126 MEK inhibitor was added at different time points during the infection and together with BDNF to abolish Arc induction. U0126 1 hbi: 1 h before infection; U0126 + HSV-1: added at the same time; U0126 1, 3, 5, 7, hpi, and HSV-1 at 8 h without inhibitor. Statistical significance was determined by One-Way ANOVA, followed by Tukey’s test. Bars represent the mean ± SD of biological replicates. Different letters above the mean bars apply to significant differences between groups *p* < 0.05. The blots are representative of three independent experiments.

Independent of the duration of U0126 treatment infected neurons showed a significant increase in Arc compared to the mock control (Figure 8A). Likewise, PSD-95, Drebrin, and CaMKIIβ showed significantly decreased expression compared to the mock control in all HSV-1 and U0126-treated neurons (Figures 8B–D). While PSD-95 showed a slight phenotype reversal when U0126 was applied 3 hpi (Figure 8B), all the alterations linked to HSV-1 infection took place regardless of the inhibition of the MEK pathway. Autophosphorylation of CaMKIIβ at T287 was also reverted when the inhibitor was applied 3 hpi (Figure 8D upper panel). When U0126 was added to neurons 7 hpi, the immunodetection for phospho-T287 CaMKIIβ was significantly higher compared to the controls (Figure 8D bottom panel). This data suggests that the Erk1/2 pathway is not the main signaling cascade involved in the Arc increase and reduction of PSD-95, Drebrin, and CaMKIIβ during HSV-1 acute infection.

The levels of Erk1/2 and CREB activation were also evaluated. The previously observed decrease in phospho-Erk1/2 was partly counteracted when U0126 was added at 1, 5, and 7 hpi, but there was no significant difference with the mock control (Figure 8E). By contrast, phosphorylation levels of CREB at S133 were reversed when the drug was added at the same time with HSV-1 (U0126 + HSV-1; Figure 8F). This result indicates that the early stage of the HSV-1 replicative cycle may be a key point regarding the alterations in the protein expression levels shown above. Further studies are required to elucidate which stage of the HSV-1 replicative cycle is responsible for the alterations mentioned in this study.

## Discussion

The present study shows that cortical neurons undergoing HSV-1 infection, display extensive loss of dendritic spines and retraction of secondary dendrites. At the protein level, an increase in Arc protein occurs concomitantly with decreased expression of the dendritic proteins involved in the maintenance of spine architecture. Also, we found that HSV-1 infected neurons are unresponsive to glutamate stimulation. The major molecular and functional alterations are: (i) highly increased Arc expression, (ii) reduced expression of post synaptic density scaffolding proteins; PSD-95, Drebrin and CaMKIIβ, and (iii) no response to glutamate stimulation. Furthermore, HSV-1-induced Arc transcription and protein expression, in contrast to mechanisms in synaptic plasticity and learning, does not depend on Erk activation. The specificity of HSV-1 as the causal agent of these alterations requires further investigation since many of the observed phenomena could be early neurodegeneration events.

In a recent report we demonstrated the induction and altered distribution of Arc protein during HSV-1 infection in several neuronal cell lines; HT22: mouse hippocampal neurons, SH-SY5Y human neuroblastoma and H4: human neuroglioma. Our findings strongly suggest that the pathogenicity of HSV-1 neuronal reactivations in humans could be mediated in part by Arc upregulation (Acuña-Hinrichsen et al., 2019). Here we show that HSV-1-infected cortical neurons exhibit spine loss and retraction of dendritic arbors, accompanied by high levels of Arc protein that is concentrated in neuronal somata, along with a loss of key structural components of dendritic spines: PSD-95, Drebrin, and CaMKIIβ.

Arc is a flexible protein and has numerous binding partners indicating its role as a hub protein (Myrum et al., 2015; Nikolaienko et al., 2018). Here we focused on a set of known Arc interaction partners: PSD-95, Drebrin and CaMKIIβ found in postsynaptic dendrites and spines to characterize the spine architecture during HSV-1 infection. Arc is found in the postsynaptic density where it forms complexes with PSD-95 and other proteins (Fernández et al., 2017). An abnormal elevation of Arc impairs PSD-95-TrkB association and signaling (Cao et al., 2013). Arc is also recruited to inactive synapses by the unphosphorylated form of CaMKIIβ in a process called inverse synaptic tagging (Okuno et al., 2012), and newly synthesized SUMOylated Arc forms a complex with Drebrin, a major regulator of cytoskeletal dynamics in dendritic spines during *in vivo* LTP (Nair et al., 2017).

These interactions reflect the diversity of Arc signaling. At the outset, we hypothesized that Arc protein induced in response to HSV-1 infection may have altered or biased protein interactions and signaling. We found that all the protein interactions were abolished despite strong increases in Arc expression. However, we found that HSV-1 infection also results in extensive progressive disruption of cortical neuronal dendritic structure. This process starts early in infection, where the total levels of these partner proteins decrease. This is not so rare for HSV-1, since infection inhibits host transcription and RNA splicing, thereby interrupting the supply of host mRNA to the cytoplasm (B Taddeo et al., 2004). Accelerated degradation of cellular proteins by post-translational modifications initiated by phosphorylation of cellular proteins by the viral protein kinases has also been described (Liang and Roizman, 2008). Therefore, we were forced to ask ourselves the question: why is only Arc up-regulated during acute HSV-1 infection? Since SARE (Synaptic Activity Response Element) implies up-regulation of Arc after synaptic activity within minutes of stimulation, and Arc accumulation in infected neurons starts at 4 hpi and peaks after 8 hours, it is reasonable to hypothesize that Arc expression is not mediated by this element alone. In this regard, UL23 is one of the most studied HSV-1 β genes (early genes) regarding its transcriptional regulatory elements and was used for the comparison with the Arc promoter. HSV-1 β-genes are characterized by the presence of eukaryotic consensus sequences, e.g., GC- box, CCAT-box and TATA box, upstream of the transcription start-site. All these transcription factor-binding sequences were also found in the Arc promoter (Supplementary Figures 3A–D). It is important to clarify that our results are not sufficient to give any causality of increased Arc protein levels to the decrease of its binding partners. More studies are necessary to better understand this process.

Neuronal calcium levels are implicated in neurite spine formation, growth cone turning, and gene transcription. The spontaneous Ca^2+^ transients are mediated by NMDA receptors and N-type VDCCs (Zheng and Poo, 2007), which may be activated by the ambient endogenous glutamate in the extracellular space. These Ca^2+^ signals activate downstream Ca^2+^ effector enzymes, leading to changes in the number and properties of postsynaptic transmitter receptors and/or in presynaptic efficacy in transmitter secretion (see Malenka and Bear, 2004). Ca^2+^ signals also trigger actin cytoskeleton rearrangement in postsynaptic spines, leading to a modified synaptic morphology. It is well known that HSV-1 promotes Ca^2+^ mediated APP phosphorylation and Aβ accumulation in cortical neurons (Piacentini et al., 2011). Our data also suggests that HSV-1 infected neurons might have altered calcium levels as shown in previous reports (Cheshenko et al., 2003, 2013). However, when we stimulated these neurons with glutamate, the relative Ca^2+^ levels did not vary, suggesting that the structural neuritic damage also affects the assembly and maintenance of receptors in the spines.

Our data also indicates that Arc over-expression could be directed by CREB activation during HSV-1 neuronal infection. The common downstream point of several surface receptors involved in mediating Arc transcription is Erk kinase, which activates transcription factors interacting with regulatory elements within the Arc gene. A SARE which contains binding sites for CREB, SRF, and MEF is enough to significantly boost Arc transcription. Nevertheless, HSV-1 does not have an impact on Erk1/2 activation before Arc induction, suggesting that there must be another kinase involved in CREB activation. A good candidate could be the stress-related p38 MAPK, since its immediate-early gene expression is sufficient for the activation of both p38 and JNK in a viral infection context (Hargett et al., 2005).

There is rising epidemiological and experimental findings in the last decades which give evidence about the causality of HSV-1 neuronal infection in Alzheimer’s disease (AD) (Letenneur et al., 2008; de Chiara et al., 2010; Itzhaki, 2018). One of the alterations of our particular interest, is the fact that HSV-1 promotes Ca^2+^ mediated APP phosphorylation and Aβ accumulation in rat cortical neurons (Piacentini et al., 2011). Viral entry induces membrane depolarization which is sustained up to 12 hpi. These effects depend on persistent sodium current activation and potassium current inhibition. The virally induced hyperexcitability triggered that significantly increased intraneuronal Ca^2+^ levels (due to both Ca^2+^ entry through Cav1 channels and Ca^2+^ release from IP_3_ receptors). That could be a possible explanation for the high Ca^2+^ levels we detected in ongoing activity in late infected neurons (either at 8 or 18 hpi). However, the HSV-1 infected hyperexcited neurons are also devoid of an appropriate structure to maintain the glutamate receptors anchored to post synaptic density. Therefore, it is logical to think that if there is no suitable structure, there is no proper synapse, and consequently no entry of extracellular Ca^2+^ when glutamate is added.

The intracellular Ca^2+^ elevation during late HSV-1 neuronal infection, and subsequent APP phosphorylation and Aβ accumulation were dependent on glycogen synthase kinase (GSK3) activation (Piacentini et al., 2015a). GSK3α/β are serine-threonine kinases, that like Arc protein, contribute to synaptic plasticity and the structural plasticity of dendritic spines. GSK3 activation was found to phosphorylate Arc protein and promote its degradation under conditions that induce *de novo* Arc synthesis. This GSK3 phosphorylation-dependent ubiquitination of residue K136, is one example of positive cross-talk between post-translational modifications of Arc protein (Gozdz et al., 2017). K24, K33, and K55 can also be acetylated, protecting Arc from ubiquitin-dependent degradation and thus stabilizing Arc half-life (Lalonde et al., 2017). This might be a stimulating idea to test, whether Arc is acetylated at 8 hpi, and therefore accumulated. There is also the possibility of Arc oligomerization under high up-regulation during HSV-1 infection (Supplementary Figure 1) due to the known low solubility of Arc oligomers (Myrum et al., 2015; Hallin et al., 2018; Maria Steene Eriksen et al., 2019). In 2013, Naghavi and colleagues demonstrated that Us3, a viral Ser/Thr kinase deactivates GSK3 at ∼9 hpi, through phosphorylation of Ser9 (Naghavi et al., 2013). Inactive GSK3 enhances the inhibitory phosphorylation of CREB at Ser129, in agreement with the significant reduction of phosphorylation at Ser133 that we showed in Figure 7B. As reported previously, the inhibition of GSK3β promotes Arc accumulation (Gozdz et al., 2017).

**FIGURE 9:**
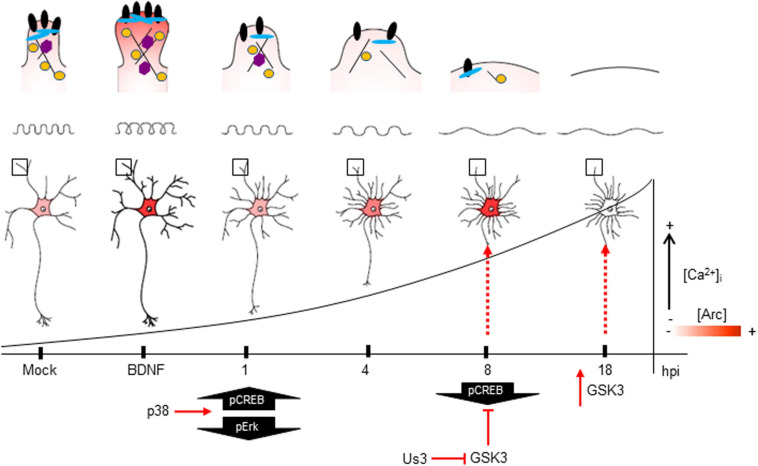
The impact of HSV-1 neuronal infection at the dendritic spine level. Diagram of the HSV-1-induced phenotype in cortical neurons at 1, 4, 8 and 18 hpi. Changes in shape and structure both in somata and dendrites are shown. Early in infection, 1 hpi CREB is activated (pS133) by p38 MAPK (Hargett et al., 2005) instead of Erk. From 1 hpi there is a progressive decrease of PSD-95, Drebrin and CaMKIIβ along the infection kinetics (upper panel zoom dendrites), most likely due to VHS viral protein in charge of downregulating the host cell mRNAs (Brunella Taddeo et al., 2007). From 4 hpi there is a notorious increase of Arc expression (shown as red color), reaching its highest level at 8 hpi. At this time, CREB activation is downregulated, most likely due to the inhibitory phosphorylation of GSK3 at S129. The viral kinase Us3 phosphorylates GSK3 at inhibitory residue (S9) (Wagner and Smiley, 2009), thus promoting Arc accumulation (Gozdz et al., 2017) (first dashed red arrow). Then, at 18 hpi, the Us3 effect is abolished and GSK3 is activated due to the rising levels of intracellular calcium (Piacentini et al., 2011). The activation of GSK3 promotes Arc degradation at late infection (18 hpi) (second dashed red arrow), this rapid turnover of Arc expression is accompanied by high S206 phosphorylation levels, which could have an impact on protein localization.

Altogether, the present work provides evidence that HSV-1 neuronal infection resembles many of the phenotypes induced by neurodegenerative diseases, such as spine loss and synaptic protein down regulation, accumulation of Arc protein, and abnormal responses to glutamate stimulation (Figure 9). The robust changes caused by HSV-1 are most likely at the end of the replicative cycle (∼ 18 hpi). However, these are normal alterations that neurons undergo when subjected to experimental-non-physiological MOIs, where apoptotic processes become inevitable. In normal physiological periodic reactivations in the brains of infected individuals, these reactivations are more focused, and there is no information available on how HSV-1 in this context affects neuronal function and fate.

Arc was discovered in 1995 due to robust and transient enhancement of its mRNA in the hippocampus of rats which had been given electrically induced seizures *in vivo* (Link et al., 1995; Lyford et al., 1995). Since then, several behavioral studies involving learning and memory tasks have shown the importance of Arc induction (Plath et al., 2006). However, over-expression of Arc caused by some drugs causes impairment in exploratory behavior (Díaz-Hung et al., 2018), as well as synaptic downscaling (Nikolaienko et al., 2018); highlighting the importance of the tight regulation of Arc expression. Since this is the first report on Arc protein dynamics and protein interactions during viral infection, it is tempting to speculate whether HSV-1-induced Arc has a negative influence on neuronal plasticity. The infected, still-surviving neurons may exhibit synapse dysfunction and aberrant behavior in their neuronal networks, which are major determinants of many neurological diseases (Paula-Lima et al., 2013).

## Materials and Methods

### Primary Cortical Neuron Cultures

Primary cortical neuron cultures were prepared according to previously described methods with several modification for this study (Ishizuka et al., 2014; Ishizuka and Bramham, 2020). Briefly, time mated pregnant Wistar rats were deeply anesthetized and sacrificed. Cortical tissues were dissected from the fetuses at embryonic day 18. The cortical tissues were treated with 0.05% Trypsin-EDTA solution (Life Technologies, Carlsbad CA) for 10 min at 37°C. Then, cells were mechanically dissociated using a polished glass pipet. The cell suspensions were seeded at 10,000-15,000 cells/cm2 on 18 mm cover glasses (Marienfeld-Superior, Lauda-Königshofen, Germany) coated with 1 mg/ml poly-L-lysine (PLL; Sigma-Aldrich, St. Louis, MO, United States) in 12 well plate for immunocytochemistry, or 40,000–60,000 cells/cm2 on 6 cm culture dishes coated with 0.1 mg/ml PLL for biochemistry, respectively. The cells were maintained in Minimum Essential Medium (MEM; Life Technologies) supplied with 10% fetal bovine serum (Sigma-Aldrich), 0.6% glucose (Sigma-Aldrich), and 1mM sodium pyruvate (Life Technologies) for 2 h. After attachment of the cells, the medium was replaced with Neurobasal^TM^ Medium (Life Technologies) containing 2% B-27^TM^ supplement (Thermo Fisher) and 0.25% GlutaMAX^TM^-I (Life Technologies). After the cells were incubated for 14 to 21 days *in vitro* (DIV), they were subjected to viral and pharmacological experiments, and then analyzed biochemically and immunocytochemically.

### HSV-1 Acute *in vitro* Infection

Herpes simplex virus type 1 (strain F) used in this study was kindly supplied by Dr. Bernard Roizman, Northwestern University, Chicago, IL, United States. The virus stocks were prepared and titrated from infected Vero cells (Ejercito et al., 1968). Infection was carried out at a multiplicity of infection (MOI) of 10 for Western blot and 5 for immunocytochemistry experiments. The virus was allowed to adsorb for 1 hour in a low volume of medium supplemented with B-27 for primary cultures, with regular mixing. Following infection, viruses were removed by aspiration, cells washed once with maintenance medium and finally, the previous normal media added. HSV-1 and mock-infected cells were further cultivated for different periods (15 min, 30 min, 1, 4, 6, 8, and 18 hpi). BDNF (100 μg/ml, 2 h incubation) was used as a positive control to Arc induction. TTX (2 μM, 2 h incubation) was used as a negative control of Arc expression. U0126 (10 μM, 1-h incubation) was used to inhibit MEK/ERK.

### Immunocytochemistry

Non-infected neurons (Mock) and HSV-1 infected neurons were fixed in 4% paraformaldehyde or ice-cold Methanol in PBS 1X for 20 min, or 5 min, respectively. Then washed in PBS 1X three times, and permeabilized in 0.2% Triton X-100 in PBS 1X for 10 min (only the PFA fixed cells). Cells were incubated 30 min at 37°C with the following primary antibodies: against Drebrin, PSD-95, CaMKIIβ, Arc, MAP-2 (Table 1) and Phalloidin probe against fibrillar actin (#10656353, Alexa Fluor^TM^, Invitrogen^TM^). Finally, cells were incubated with the corresponding secondary antibodies conjugated with Alexa-488 (#A32723, Goat anti-Mouse IgG (H+L) Highly Cross-Adsorbed Secondary Antibody, Alexa Fluor Plus 488) or Alexa-568 (#A-21090, Goat anti-Human IgG (H+L) Cross-Adsorbed Secondary Antibody, Alexa Fluor 568). Fluorescence images were obtained from 10 cells per treatment using a Zeiss Axioskope A1 epifluorescence microscope (Carl Zeiss, Göttingen, Germany) with a digital video camera (Nikon DXM 1200). The images obtained (at least ten microscopic fields per each sample) were processed using Image J software (National Institutes of Health).

**TABLE 1:** Key resources table.

**Reagent or Resource**	**Source**	**Identifier**
**Antibodies**		
Rabbit polyclonal anti-Arc	Synaptic Systems	Cat# 156003
Mouse monoclonal anti-ICP8	Santa Cruz Biotechnology	Cat# sc-53329, clone10A3
Mouse monoclonal anti-Drebrin	Abcam	Cat# ab-12350, clone M2F6
Mouse monoclonal anti-PSD95	Thermo Fisher Scientific	Cat# MA1-046, clone 7E3-1B8
Mouse monoclonal anti-CaMKIIβ	Thermo Fisher Scientific	Cat# 139800, clone CB-beta1
Rabbit polyclonal anti-phospho-CaMKIIβ/γ/δ (Thr287)	Thermo Fisher Scientific	Cat# PA5-39731
Rabbit polyclonal anti-Total Erk1/2	Cell Signaling Technology	Cat# 4695S
Rabbit polyclonal anti-phospho Erk1/2(MAPKpT202/pY204	Cell Signaling Technology	Cat# 9101S
Rabbit monoclonal anti-CREB	Cell Signaling Technology	Cat# 9197, clone 48H2
Rabbit monoclonal anti-phospho CREB Ser133	Cell Signaling Technology	Cat# 9198, clone 87G3
Rabbit polyclonal anti-HSV-1/2 total proteins	Abcam	Cat# ab9533
Mouse monoclonal anti-GAPDH	Thermo Fisher Scientific	Cat# MA5-15738, clone GA1R
Mouse monoclonal anti-β-Actin	Sigma Aldrich	Cat# A5441, clone AC-15
Chicken polyclonal anti-MAP-2	Encor Biotechnology	Cat# CPCA-MAP2
Alexa-488, Goat anti-Mouse IgG (H+L) Highly Cross-Adsorbed Secondary Antibody, Alexa Fluor Plus 488.	Thermo Fisher Scientific	Cat# A32723
Alexa-568, Goat anti-Human IgG (H+L) Cross-Adsorbed Secondary Antibody, Alexa Fluor 568.	Thermo Fisher Scientific	Cat# A-21090
**Bacterial and Virus Strains**		
HSV-1 (strain F)	supplied by Dr. Bernard Roizman, Northwestern University, Chicago, United States.	
**Chemical, Peptides and Recombinant Proteins**		
Trypsin-EDTA (0.05%), phenol red	Thermoscientific	Cat# 25300054
Poly-L-lysine hydrobromide	Sigma Aldrich	Cat# P2636-500MG
MEM medium	Thermoscientific	Cat# 11095080
Fetal bovine serum	Sigma Aldrich	Cat# 1 F7524
D-Glucose	Sigma Aldrich	Cat# G8769
Sodium Pyruvate (100 mM)	Thermo Fisher Scientific	Cat# 11360070
Neurobasal medium	Thermo Fisher Scientific	Cat# 21103049
Gibco^TM^ B-27^TM^ Supplement, Serum Free, 10 mL	Thermo Fisher Scientific	Cat# 11530536
GlutaMAX	Life Technologies	Cat# 35050-038
BDNF	Alomone	Cat# B-250
Tetrodotoxin citrate, Na+ channel blocker	Abcam	Cat# ab120055
U0126 monoethanolate	Sigma Aldrich	Cat# U120
Triton X-100	Sigma Aldrich	Cat# T8787
Pierce^TM^ 16% Formaldehyde (w/v), Methanol-free	Thermo Fisher Scientific	Cat# 11586711
Phalloidin	Thermo Fisher Scientific	Cat# 10656353
RIPA buffer 1X	Sigma Aldrich	Cat# R0278
Protease and Phosphatase inhibitors	Sigma Aldrich	Cat# 11836170001

Sholl analysis was performed with a semiautomated program, in which the somata boundary is approximated by an ellipsoid and dendrite intersections were assessed at radial distances from the somata (Charych et al., 2006). The dendritic tree was examined in 10 μm increments. The dendritic spine density was analyzed by ImageJ software as well as the dendritic spine density. Statistical analysis was done with ANOVA followed by the appropriate post hoc test.

### Immunoblot

For biochemical analysis, neurons from different treatments were harvested and lysed in RIPA buffer 1X (#R0278, Sigma-Aldrich Corporation, St Louis, MO, United States) supplemented with protease and phosphatase inhibitors (#11836170001, Sigma-Aldrich Corporation St Louis, MO, United States) and the protein concentration was quantified with Micro BCA^TM^ Protein Assay Kit (Thermo Fischer Scientific, Waltham, MA United States). Equal amounts of protein (20 μg per line) were loaded and separated by SDS–PAGE (10% polyacrylamide) and transferred to PVDF membrane (#L-08007-001, AH-Diagnostic) previously activated with methanol. The membranes were incubated at room temperature in blocking solution (3% BSA in TBS-Tween 0,1%), and then incubated overnight with the primary antibodies listed in Table 1, diluted in TBST-3% BSA. Next, they were incubated with appropriate secondary antibodies (anti-rabbit and anti-mouse from Thermo Fischer Scientific, Waltham, MA United States). Following three washes with TBST, blots were incubated for 1 h at RT in horseradish peroxidase-conjugated secondary antibody diluted in TBST. Blots were then visualized using enhanced chemiluminescence (ECL Western Blotting Substrate; Pierce). The films were scanned, and the resulting images were analyzed by densitometry to determine the relative levels of each protein, using the Un-Scan-IT gel 6.1 software.

### Isolation of Synaptoneurosomes

Adapted from Villasana et al. (2006), with some modifications. Briefly, cortical neurons mock-infected and HSV1-infected were homogenized in synaptoneurosome buffer (10 mM HEPES, 1 mM EDTA, 2 mM EGTA, 0.5 mM DTT, 10 μg/ml leupeptin, and 50 μg/ml soybean trypsin inhibitor, pH 7.0) at 4°C using cell scrappers. A fraction of homogenate sample was set aside for Western blot analysis. From this step forward the homogenate was always kept ice-cold to minimize proteolysis throughout the isolation procedure. The sample was loaded into a 60 ml Leurlok syringe and filtered twice through three layers of a prewetted 100 μm pore nylon filter held in a 13 mm diameter filter holder. The resulting filtrate was then loaded into a 5 ml Leurlok syringe and filtered through a pre-wetted 5 μm pore hydrophilic filter held in a 13 mm diameter filter holder. Because the 5 μm pore size filter produces more pressure and requires changing out the filter within samples, smaller 5 ml volumes were filtered to keep the sample cold while filtering, and then were pooled together. The resulting filtrate was placed in a 50 ml polycarbonate tube and centrifuged at 1000 × *g* for 10 min. The pellet obtained corresponded to the synaptoneurosome fraction. Isolated synaptoneurosomes were resuspended in 100 μl of RIPA buffer.

### Immunoprecipitation

To immunoprecipitate Arc from the synaptoneurosomal fraction, we used PureProteome^TM^ Protein A/G Mix Magnetic Beads (Millipore, Cat # LSKMAGS08) following the manufacturer’s Protocol B instructions. Briefly, 1 μg/μl of Arc and IgG antibodies (Table 1) were incubated with magnetic beads in PBS 1X -0.01% Tween 20 for 45 min at RT with 1000 rpm agitation. Then, the Antibody-Bead complex was washed three times in PBS 1X -0,01% Tween 20 and then incubated with the synaptoneurosomal fraction total lysate, previously quantified, for 4 hours at −20°C in constant agitation. Next, the samples were washed three times with mild lysis buffer (M-RIPA: 50 mM Tris-HCl; 150 mM NaCl; 1% NP-40; 0.25% sodium deoxycholate, 1 mM EDTA), and two times with PBS 1X -0.1% Tween 20. Finally, we added sample buffer and incubated the samples for 10 min at 70°C, rescued the supernatant and then Arc precipitates were analyzed by Western Blot.

### Calcium Imaging

Cortical neurons were grown on glass slides to then undergo infection the day of the experiment. The primary culture medium was replaced with a Ca^2+^-containing HEPES buffered salt solution (IB Buffer) composed of (mM): 15 Hepes (pH 7.4), 135 NaCl, 5 KCl, 1.8 CaCl_2_, 0.8 MgCl_2_ supplemented with 20 mM glucose. Neurons were then incubated with 5 μM Fluo-4-AM for 15 min before starting recordings. Then, they were placed in an open-bath imaging chamber containing IB Buffer, where the basal Ca^2+^ levels were registered for about 6 min before starting the stimulation. The glycine/glutamate stimulation was carried out using 0.1 mM Glycine and seconds later 0.5 mM Glutamate (Calbiochem). An additional control includes cells exposed to 1 μM of ionomycin (Thermofisher) at the end of the experiment since it induces pores in the plasma membrane, increasing Ca^2+^ entry into the cell. Imaging of local cytosolic Ca^2+^ levels was accomplished using a confocal laser scanning microscope, and neurons were excited at 488 nm, and the emission fluorescence was collected using a 505–525 nm filter. Images were processed and analyzed with ImageJ software where first the background was subtracted from the image stack. The fluorescence intensity of the ROIs was normalized for each area, and the values were then plotted against time and shown as (F-F_0_)/F_0_, where F_0_ corresponds to the mean fluorescence intensity during the first 50 s (Covarrubias-Pinto et al., 2020).

### Statistical Analysis

All the results are representative of at least three independent experiments. Results were analyzed by one-way or two-way analyses of variance (ANOVA) followed by the appropriate post-test using GraphPad Prism v.6 software. The data were expressed as means ± standard deviations. The value of *p* < 0.05 was regarded as statistically significant and indicated in the figure in different letters above bars mean.

## Data Availability Statement

The original contributions presented in the study are included in the article/Supplementary Material, further inquiries can be directed to the corresponding author/s.

## Ethics Statement

All animal procedures were performed in accordance with the Bioethical of the Universidad Austral de Chile and the Norwegian animal care committee regulations.

## Author Contributions

CO, MAC, CRB, and FA-H conceived and designed the experiments, analyzed the data, and wrote the article. FA-H, AC-P, YI, CM, and PS performed the experiments. MAC, CO, CRB, and MS contributed reagents, materials, and analysis tools. All authors contributed to the article and approved the submitted version.

## Conflict of Interest

The authors declare that the research was conducted in the absence of any commercial or financial relationships that could be construed as a potential conflict of interest.

## Supplementary Material

Supplementary Figure 1 Presence of high molecular weight Arc complex; comparison between Arc overexpression in cortical and hippocampal HSV-1 infected neurons. **(A)** Immunoblot analyses of ICP8, Arc, HSV-1 total proteins and GAPDH as a loading control. **(B)** Quantification of densitometries of Arc (55 kDa) and C HSV-1, respectively. The arrow shows high molecular weight oligomers in Arc immunodetection panel (∼150 kDa). The blots are representative of three different experiments and were analyzed by Two-way ANOVA and Bonferroni post-hoc test for multiple comparisons. ^∗∗∗^*p* < 0.001; ^∗∗^*p* < 0.01; ^∗^*p* < 0.05; n.s. = non-significant.

Supplementary Figure 2 HSV-1 infection induces CREB phosphorylation and nuclear translocation in hippocampal cell lines. An HT22 mouse hippocampal cell line was infected with HSV-1 MOI 10, at 1, 4, 6, 8, and 18 h. **(A)** Immunoblot analyses of total protein extracts show an increase in phosphorylation of CREB at S133 residue, starting from 1 hpi compared with mock-infected cells. **(B)** Immunohistochemistry analyses of mock-infected and 4 hpi cells, stained against phospho-S133-CREB (green), ICP8 (red), and DAPI for nuclei (blue). **(C)** CREB mRNA relative expression levels at Mock, 4 and 8 hpi.

Supplementary Figure 3 Arc and HSV-1 TK genes share common transcriptional regulatory elements. *In silico* analyses of Arc **(A)** and UL23 **(B)** gene promoter show the presence of C CAAT-box **(C)**, GC-box **(D)**, and TATA-box **(E)** consensus sequences. The location of every site is depicted regarding the transcription start site (+1).
