# Supplementary figures and images for: Herpes Simplex Virus Type 1 Neuronal Infection Triggers the Disassembly of Key Structural Components of Dendritic Spines
Source: Front Cell Neurosci. 2021 Feb 23;15:580717. doi: 10.3389/fncel.2021.580717 (PMC7940845; doi:10.3389/fncel.2021.580717)

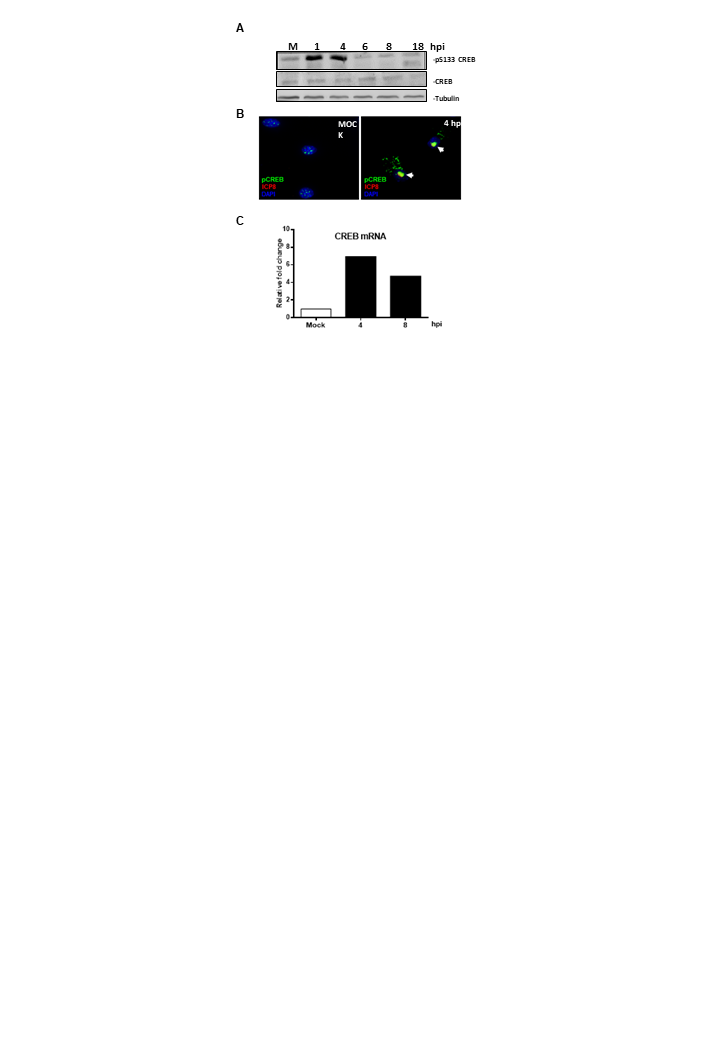

Supplement: Supplementary Figure 1 — Presence of high molecular weight Arc complex; comparison between Arc overexpression in cortical and hippocampal HSV-1 infected neurons. (A) Immunoblot analyses of ICP8, Arc, HSV-1 total proteins and GAPDH as a loading control. (B) Quantification of densitometries of Arc (55 kDa) and C HSV-1, respectively. The arrow shows high molecular weight oligomers in Arc immunodetection panel (∼150 kDa). The blots are representative of three different experiments and were analyzed by Two-way ANOVA and Bonferroni post-hoc test for multiple comparisons. ∗∗∗p < 0.001; ∗∗p < 0.01; ∗p < 0.05; n.s. = non-significant. [file Image_1.tif]

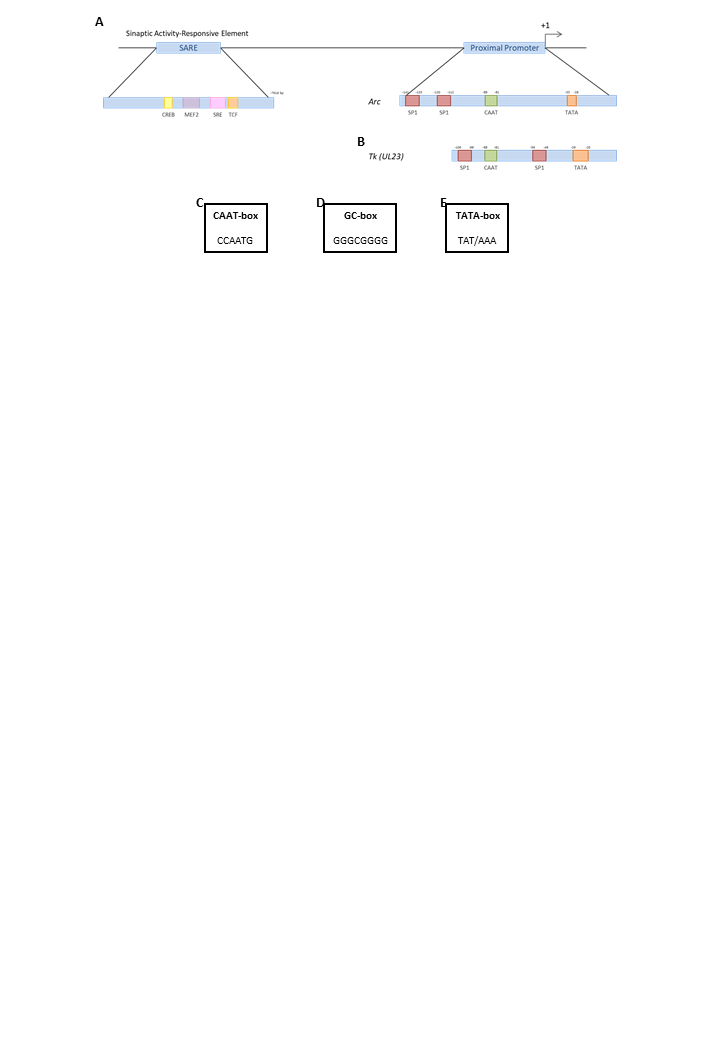

Supplement: Supplementary Figure 2 — HSV-1 infection induces CREB phosphorylation and nuclear translocation in hippocampal cell lines. An HT22 mouse hippocampal cell line was infected with HSV-1 MOI 10, at 1, 4, 6, 8, and 18 h. (A) Immunoblot analyses of total protein extracts show an increase in phosphorylation of CREB at S133 residue, starting from 1 hpi compared with mock-infected cells. (B) Immunohistochemistry analyses of mock-infected and 4 hpi cells, stained against phospho-S133-CREB (green), ICP8 (red), and DAPI for nuclei (blue). (C) CREB mRNA relative expression levels at Mock, 4 and 8 hpi. [file Image_2.tif]

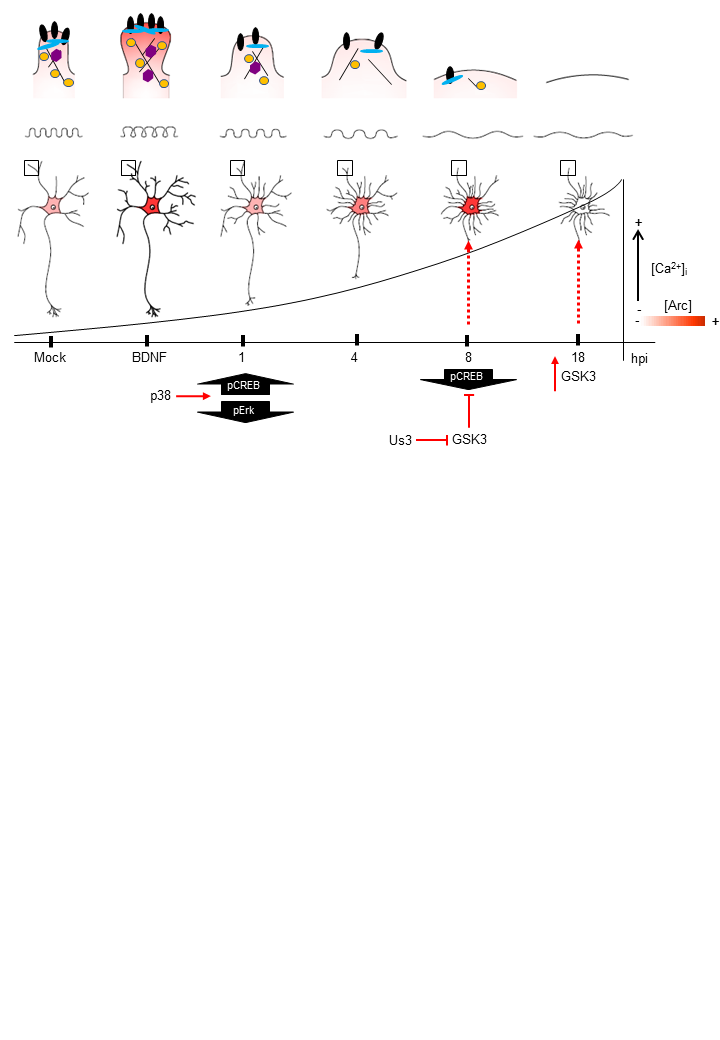

Supplement: Supplementary Figure 3 — Arc and HSV-1 TK genes share common transcriptional regulatory elements. In silico analyses of Arc (A) and UL23 (B) gene promoter show the presence of C CAAT-box (C), GC-box (D), and TATA-box (E) consensus sequences. The location of every site is depicted regarding the transcription start site (+1). [file Image_3.tif]
